# Midline catheter use for cancer patients receiving 5-FU chemotherapy: prospective study of safety and outcomes

**DOI:** 10.1093/oncolo/oyaf108

**Published:** 2025-05-27

**Authors:** Tomoki Sakakida, Shinichiro Fukahori, Taro Mizuno, Yasunobu Ishizuka, Munehiro Wakabayashi, Hiroyuki Kodama, Yukiya Narita, Toshiki Masuishi, Kazunori Honda, Shigenori Kadowaki, Masashi Ando, Kei Muro, Akinobu Ogawa, Chiho Kudo, Isao Oze, Hiroya Taniguchi

**Affiliations:** Department of Clinical Oncology, Aichi Cancer Center Hospital, Nagoya, 464-8681, Japan; Department of Nursing, Aichi Cancer Center Hospital, Nagoya, 464-8681, Japan; Department of Clinical Oncology, Aichi Cancer Center Hospital, Nagoya, 464-8681, Japan; Department of Clinical Oncology, Aichi Cancer Center Hospital, Nagoya, 464-8681, Japan; Department of Clinical Oncology, Aichi Cancer Center Hospital, Nagoya, 464-8681, Japan; Department of Clinical Oncology, Aichi Cancer Center Hospital, Nagoya, 464-8681, Japan; Department of Clinical Oncology, Aichi Cancer Center Hospital, Nagoya, 464-8681, Japan; Department of Clinical Oncology, Aichi Cancer Center Hospital, Nagoya, 464-8681, Japan; Department of Clinical Oncology, Aichi Cancer Center Hospital, Nagoya, 464-8681, Japan; Department of Clinical Oncology, Aichi Cancer Center Hospital, Nagoya, 464-8681, Japan; Department of Clinical Oncology, Aichi Cancer Center Hospital, Nagoya, 464-8681, Japan; Department of Clinical Oncology, Aichi Cancer Center Hospital, Nagoya, 464-8681, Japan; Department of Nursing, Aichi Cancer Center Hospital, Nagoya, 464-8681, Japan; Clinical Research Support Section, Aichi Cancer Center Hospital, Nagoya, 464-8681, Japan; Division of Cancer Epidemiology and Prevention, Aichi Cancer Center Research Institute, Nagoya, 464-8681, Japan; Department of Clinical Oncology, Aichi Cancer Center Hospital, Nagoya, 464-8681, Japan

**Keywords:** midline catheter, phlebitis, 5-FU, chemotherapy

## Abstract

**Background:**

Peripheral intravenous 5-fluorouracil (5-FU) administration often causes phlebitis and necessitates catheter replacement, imposing burdens on both patients and healthcare providers. Insertion of a midline catheter (MLC) into the upper arm with tip positioned in the axillary vein may reduce the incidence of phlebitis. This study evaluated the safety and effectiveness of MLC use for continuous 5-FU infusion in cancer patients.

**Methods:**

This prospective study included patients with cancer requiring at least 4 days of continuous 5-FU infusion. The primary endpoint was the incidence of phlebitis. Secondary endpoints were the success rate of MLC insertion, complications, and patient-reported outcomes.

**Results:**

Of the 61 patients enrolled, 59 were included in the analysis. The median age was 68 years, and primary cancer types were esophageal (51%) and head and neck (46%). The median MLC indwelling duration was 5.5 days (2-28 days). No phlebitis was observed (0%, 95% CI: 0–6.2), achieving the primary endpoint. The insertion success rate was 98.3%, with complications in 6.8%. Over 90% of patients and 80% of healthcare providers reported high satisfaction levels.

**Conclusion:**

MLC insertion is a safe and effective approach for continuous 5-FU infusion, eliminating phlebitis, potentially improving patients’ quality of life, and reducing healthcare providers’ workloads. (**ClinicalTrials.gov Identifier: jRCTs042230058).**

## Discussion

Regimens involving continuous infusion of 5-fluorouracil (5-FU), widely employed for patients with esophageal and head-and-neck cancers, require administration periods of 96 hours or longer. As a high-osmolarity alkaline agent, 5-FU is an irritant drug, significantly raising the risk of phlebitis when delivered via a peripheral venous catheter (PVC) into superficial veins. This can cause pain, redness, induration, and pigmentation along the vessel from the injection site, adversely affecting patients’ quality of life (QOL). Consequently, administration via central venous devices, such as the central venous port (CV port), central venous catheter (CVC), or peripherally inserted central catheter (PICC) are recommended to minimize the risks of phlebitis and extravasation.^1-3^ However, these devices pose challenges, including serious complications such as central line-associated bloodstream infection (CLABSI) and deep vein thrombosis (DVT). Furthermore, from a cost perspective, continuous 5-FU infusion via PVC remains standard in clinical practice in Japan and some other countries.^4,5^

Use of a midline catheter (MLC), a 7.5-20-cm catheter inserted into the upper arm without fluoroscopic guidance, has led to a lower incidence of phlebitis in comparison with PVC use.^6^ This is likely due to catheter tip placement in the axillary vein, where the greater diameter and increased flow rate are expected to markedly lower the risk of 5-FU-induced chemical phlebitis. Additionally, unlike PVC, MLC does not require routine replacement, which may not only reduce patient discomfort associated with frequent venipunctures but also lessen the burden on healthcare providers caused by PVC reinsertion. While MLC use has been reported in emergency and critical care settings, there is limited evidence regarding its application in chemotherapy.

**Figure 1.**
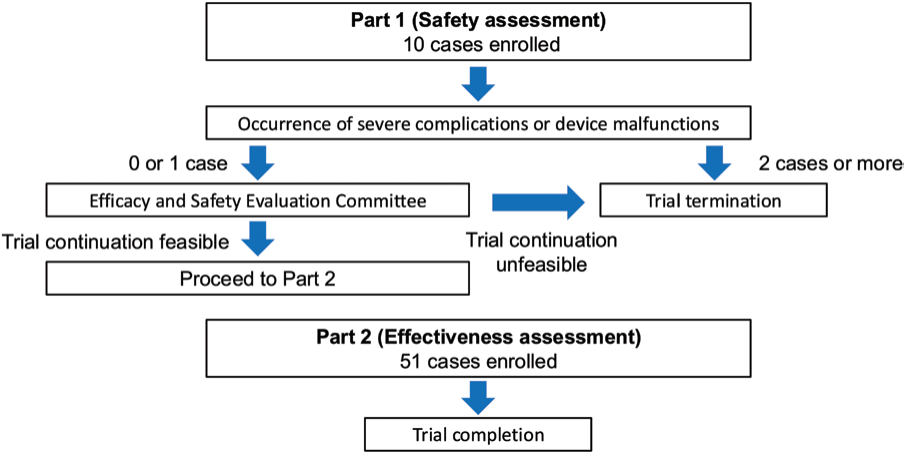
Study design. This study adopts a two-part design: Part 1 evaluates safety; Part 2 evaluates effectiveness and safety.

In this prospective study, designed in 2 parts: a safety evaluation and an effectiveness evaluation (**Figure 1**), the incidence of phlebitis associated with continuous infusion of 5-FU via MLC was 0% (95% CI: 0%-6.2%), below the predetermined threshold of 16%; thus, the primary endpoint was met. The complication rate associated with MLC insertion and indwelling was 6.8%, with one case each of catheter occlusion, catheter infection, deep vein thrombosis, and arterial puncture, consistent with prior reports. Additionally, all catheter insertions were performed by nurses who had completed specialized training, resulting in a high placement success rate of 98.3%. In the patient survey, 92.6% of participants reported that they were either “very satisfied” or “satisfied” with MLC, revealing strong patient support (for further details, please refer to Table 1 and outcome notes of primary and secondary assessment).

**Table 1.** Results of using midline catheter for continuous 5-FU infusion.

Factors	Value
Incidence of phlebitis, % (95% CI)	0 (0-6.2)
Success rate of catheter insertion, % (95% CI)	98.3 (91.0-99.7)
Incidence of complications/malfunctions on midline catheter indwelling, % (95% CI)	6.8 (2.2-16.6)
Rate of satisfaction with midline catheter, %	Very satisfied: 64.2, satisfied: 28.3

Continuous infusion of 5-FU via MLC demonstrated both high-level safety and effectiveness. Furthermore, the reduced risks of phlebitis suggest potential advantages in alleviating workloads of healthcare providers and minimizing patient discomfort, contributing to enhanced QOL of cancer patients.

## Lessons learned

- Continuous infusion of 5-FU via midline catheter use was associated with a phlebitis incidence of 0%, along with low complication rates and high-level patient satisfaction.
- The reduced risk of phlebitis suggests benefits in reducing burdens for both patients and healthcare providers, highlighting its potential for broader application in cancer treatment.

**Table:** 

Trial Information	
Disease	All solid tumors receiving 5-FU infusion over four days
Stage of disease/ treatment	No designated stage/perioperative or palliative chemotherapy
Prior therapy	No designated number of regimens
Type of study	Interventional single-arm feasibility study
Primary endpoint	Incidence of phlebitis
Secondary endpoints	Success rate of midline catheter insertion, Incidence of complications or malfunctions from midline catheter indwelling, patient-reported outcomes regarding injection site

## Additional details of endpoints or study design

IRB Approved: CRB4200002

## Study design

This interventional feasibility study followed a single arm, prospective, 2-part design and was carried out at a single institution. The two parts were as follows: Part 1 focused on verifying the safety of administering continuous intravenous infusion of 5-FU using a midline catheter; Part 2 assessed both its effectiveness and safety.

## Objective

To assess the safety and effectiveness of MLC use for continuous 5-FU infusion in cancer patients.

## Eligibility criteria

The key inclusion criteria were as follows: patients undergoing continuous 5-FU infusion for 4 days or more, aged 18 or older, with an ECOG performance status of 0-1. Patients with significant comorbidities (eg, ischemic heart disease, heart failure, interstitial pneumonia, pulmonary fibrosis, uncontrolled hypertension, or diabetes), serious infections, specific anatomical or neurological conditions impeding catheter insertion, hemostatic and coagulation abnormalities (prothrombin time ≤ 50% or INR ≥ 1.5; platelet count ≤ 50 000/mm³), or those receiving multiple antithrombotic drugs were excluded.

## Procedures

MLC was inserted by specialized, trained nurses using the 8-cm Arterial Leadercath (Vygon). Under ultrasound guidance, the basilic or brachial vein was selected, punctured, and venous backflow was confirmed before guidewire insertion. Once the guidewire had been smoothly positioned, the catheter was inserted, and the appropriateness of positioning was verified based on ultrasound and blood backflow. The catheter was removed in the same manner as standard PVC if adverse events occurred, upon completion of the planned continuous 5-FU infusion for one cycle, or after the subsequent intravenous treatment had been completed.

## Evaluation

The primary endpoint was the incidence of phlebitis among subjects enrolled in Parts 1 and 2 who underwent MLC insertion and continuous 5-FU infusion. Phlebitis was assessed according to the Phlebitis Scale as defined by the American Infusion Nurse Society.^7^

Secondary endpoints included the: success rate of MLC insertion, incidence of complications or malfunctions arising from MLC insertion and indwelling, and patient-reported outcomes regarding the injection site. The incidence of adverse events was graded according to the Common Terminology Criteria for Adverse Events (CTCAE) version 5.0. Patient-reported outcomes regarding the injection site were based on the Japanese version of PRO-CTCAE & grade version 1.0. The incidence of phlebitis, adverse events, and complications was evaluated from the time of MLC placement through to the completion of 5-FU infusion and during follow-up examinations after the completion of administration.

## Statistical analyses

Based on reports showing a phlebitis occurrence rate of 16%-86% associated with 5-FU administration via PVC,^8-10^ the required sample size was calculated using a binomial one-sample test, assuming a threshold phlebitis incidence of 16% and an expected incidence of 4%, with one-sided alpha of 0.05 and 90% power. The minimum target sample size was determined as 51 patients. Descriptive statistics included Fisher’s exact and Wilcoxon rank-sum tests. All P-values presented are two-sided, with significance set at *P*-value of.05.

**Table:** 

Drug information	
**Generic/working name**	5-Fluorouracil
**Company name**	Commercially available
**Drug type**	Antimetabolites
Drug class	Small molecule
Dose	750-1,000
**Unit**	mg/m^2^
**Route**	IV via midline catheter
Schedule of administration	750 mg/m^2^, days 1-5 (docetaxel + cisplatin + 5-FU therapy) 800 mg/m^2^, days 1-5 (5-FU + cisplatin ± nivolumab/pembrolizumab therapy) 1000 mg/m^2^, days 1-4 (5-FU + cisplatin ± cetuximab therapy)

**Figure 2.**
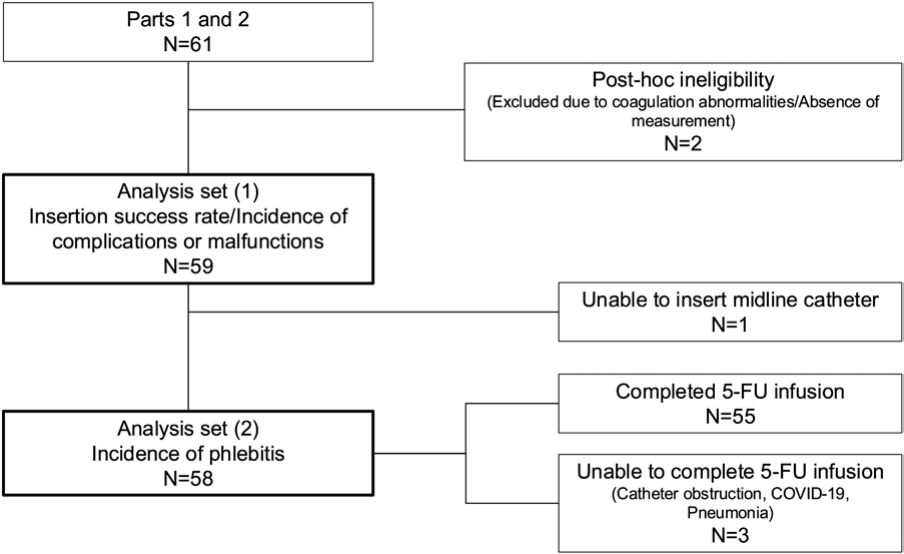
Patient flow.

Please refer to Figure 2 for patient flow.

**Table:** 

Patient characteristics	
Number of patients, male	51 (86.4%)
Number of patients, female	8 (13.6%)
Stage	Not collected
Age: median (range)	68 (39-82)
Number of prior systemic therapies: median (range)	0 (0-2): among 25 patients with palliative chemotherapy
Performance status: ECOG	0: 45 (76.2%) 1: 14 (23.8%) 2: 0 3: 0 4: 0
Cancer types or histologic subtypes	Esophageal cancer: 30 (50.8%) Head-and-neck cancer: 27 (45.8%) Urethral cancer: 1 (1.7%) Anal canal cancer: 1 (1.7%)

Please refer to Table 2 for baseline characteristics.

**Table 2.** Baseline characteristics.

Factors	*N* = 59
Age, median (range)	68 (39-82)
Male, *n* (%)	51 (86.4)
ECOG PS 0, *n* (%)	45 (76.2)
Cancer type, *n* (%)	
Esophageal	30 (50.8)
Head and neck	27 (45.8)
Urethral	1 (1.7)
Anal canal	1 (1.7)
Chemotherapy regimen, *n* (%)	
Docetaxel + Cisplatin + 5-FU	24 (40.7)
Cisplatin + 5-FU + Nivolumab/Pembrolizumab	13 (22.0)
Cisplatin + 5-FU	16 (27.1)
Cisplatin + 5-FU + Cetuximab	3 (5.1)
Others	3 (5.1)
Purpose of chemotherapy, *n* (%)	
Perioperative	29 (49.1)
Definitive	5 (8.5)
Palliative	25 (42.4)
History of chemotherapy, *n* (%)	40 (67.8)
History of phlebitis, *n* (%)	12 (20.3)
History of diabetes, *n* (%)	6 (10.2)
Use of antiplatelet/anticoagulant agents, *n* (%)	3 (5.1)

**Table:** 

Primary assessment method	
Title	Incidence of phlebitis
Number of patients screened	61
Number of patients enrolled	61
Number of patients evaluable for toxicity	59
Number of patients evaluated for efficacy	58

## Outcome notes

Among the 58 patients receiving 5-FU via MLC, 55 completed treatment, while 3 discontinued it due to catheter obstruction, COVID-19, or pneumonia. The overall incidence of phlebitis was 0% (95% CI: 0-6.2).

**Table:** 

Secondary assessment method	
Title	Success rate of MLC insertion, Incidence of complications or malfunctions from MLC indwelling, Patient-reported outcomes
Number of patients screened	61
Number of patients enrolled	61
Number of patients evaluable for toxicity	59
Number of patients evaluated for efficacy	58

## Outcome notes

**Figure 3.**
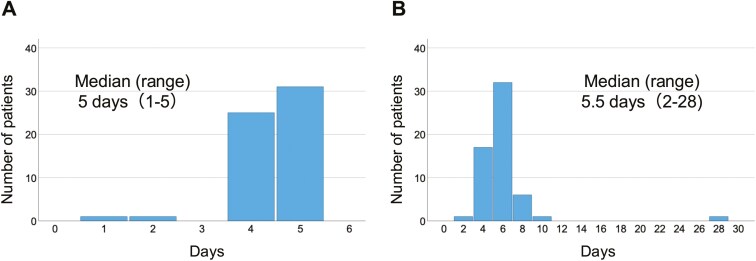
Duration of midline catheter indwelling. (A) Days under 5-FU infusion and (B) total days of indwelling.

Clinical findings during MLC insertion and the catheter indwelling duration are shown in Table 3 and Figure 3.

**Table 3.** Clinical findings during midline catheter insertion.

Factors	Value
Systolic blood pressure, mmHg (range)	119 (87-169)
Diastolic blood pressure, mmHg (range)	72 (44-108)
Pulse, bpm (range)	76 (56-100)
Vessel diameter, mm (range)	4.9 (3.2-7.8)
Vessel depth, mm (range)	4.2 (1.1-10)
Catheterized vessel (basilic/brachial)*, *n*	44/14
Catheter placement side (right arm/left arm)*, *n*	11/47
Number of punctures, *n* (range)	1 (1-3)
Procedure time, minutes (range)	4 (2–29)

* Excluding one case of unsuccessful catheter placement.

Success rate of MLC insertion was 98.3% (95% CI: 91.0-99.7).

Incidence of complications or malfunctions from MLC indwelling.

- From MLC insertion to the end of continuous 5-FU infusion: 3.4% (95% CI: 0.3-9.0), one case each of occlusion and arterial puncture
- From the end of 5-FU infusion to follow-up: 3.4% (95% CI: 0.3-9.0), one case each of deep vein thrombosis and central line-associated bloodstream infection
- Overall incidence: 6.8% (95% CI: 2.2-16.6).

**Figure 4.**
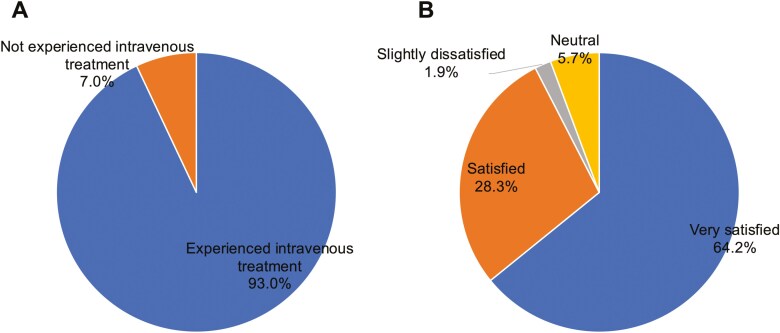
Survey results from patients. (A) Responses to whether they had prior experience with intravenous treatment. (B) Satisfaction with midline catheters compared with previous intravenous treatments. Panel A includes responses from surveyed patients (N = 57), while panel B reflects patients with prior intravenous therapy (*N* = 53).

**Figure 5.**
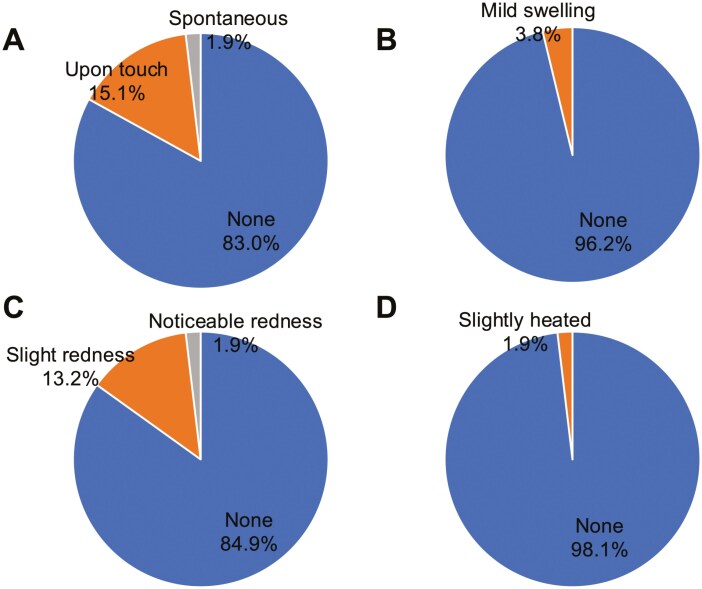
Patient-reported outcomes regarding injection sites. Each chart represents patient evaluations: (A) pain, (B) swelling, (C) redness, and (D) warmth. Each panels reflect patients with prior intravenous therapy (*N* = 53).

Patient-reported outcomes (PRO-CTCAE™ Japanese Version 1.0) regarding the injection site are shown in Figures 4 and 5 (among 57 questionnaire respondents).

Prior intravenous treatment experience: “Yes” 53 (93.0%), “No” 4 (7.0%).

Injection site symptoms (53 cases analyzed, excluding 4 without prior intravenous treatment experience):

- Pain: None, 44 (83.0%); upon touch, 8 (15.1%); spontaneous, 1 (1.9%)
- Swelling: None, 51 (96.2%); mild detectable swelling, 2 (3.8%)
- Redness: None, 45 (84.9%); slight redness, 7 (13.2%); noticeable redness, 1 (1.9%)
- Warmth: None, 52 (98.1%); slight warmth, 1 (1.9%)

Satisfaction with MLC use compared with previous infusion methods (53 cases analyzed, excluding 4 without prior intravenous treatment experience):

- Very satisfied, 34 (64.2%); satisfied, 15 (28.3%); slightly dissatisfied, 1 (1.9%); neutral, 3 (5.7%).

**Figure 6.**
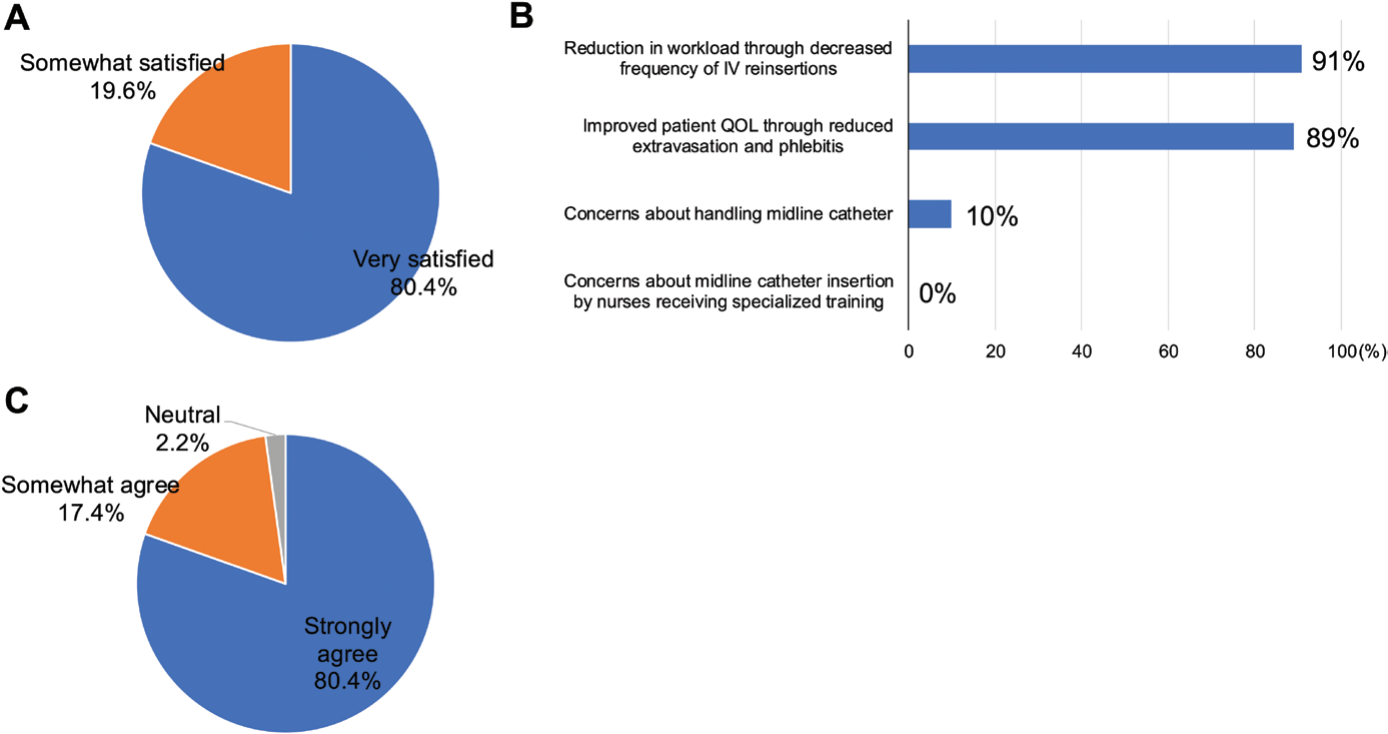
Survey results on midline catheters from 56 healthcare providers. (A) shows satisfaction with MLC compared with peripheral venous catheters, (B) outlines the reasons for satisfaction, and (C) reflects whether MLC insertion by nurses with specialized training reduced the daily workload.

**Figure 6** shows the questionnaire results on MLC use from 56 healthcare providers in this study.

## Assessment, analysis, and discussion

**Table:** 

Completion	Study completed
**Investigator’s assessment**	Active and should be pursued further

### Extended discussion

This study demonstrated that MLC insertion for continuous 5-FU infusion resulted in a markedly lower incidence of phlebitis, thereby alleviating patients’ burden and promoting their QOL. To the best of our knowledge, this is the first prospective study exploring the utility of MLC use for chemotherapy delivery.

PVC is generally safe but associated with marked complications, including high rates of insertion failure (43-59%), phlebitis (16-23%), extravasation and leakage (14-24%), accidental dislodgement (7-18%), and infection (0.44%).^11^ Phlebitis, a notable concern due to symptoms such as pain, erythema, induration, and vessel pigmentation, can negatively impact patients’ QOL. Known risk factors for phlebitis include cytotoxicity, pH (pH below 4 or above 8), and osmolarity (ratio over 2) of the drug, as well as prolonged drug contact with vessels.^12,13^ For 5-FU, an alkaline agent (pH 8.2–8.6) with a highly hyperosmolar profile (osmolarity ratio of approximately 4), PVC use has been associated with phlebitis in 16%-86% of patients. While central venous devices mitigate the risks of phlebitis and extravasation, they are associated with severe complications and higher costs. Based on these considerations, PVC remains the routinely used approach for 5-FU administration in daily clinical practice in Japan, except when suitable peripheral veins are unavailable for cannulation.

MLC is being increasingly recognized as a beneficial choice in clinical scenarios, as it can be inserted without fluoroscopic guidance, offering an accessible and convenient procedure at the bedside. The Michigan Appropriateness Guide for Intravenous Catheters (MAGIC) recommends MLC for patients with challenging vascular access, treatment durations exceeding six days, and infusions lasting up to 14 days.^14^ A large multicenter study of 10 863 patients requiring vascular access for fewer than 30 days assessed complications associated with MLC and PICC, including DVT, CLABSI, and occlusion. This study was adjusted for patient comorbidities and catheter indwelling times, revealing that MLC was significantly correlated with lower complication rates than PICC (3.9 vs. 9.9%, OR: 1.99 [95% CI: 1.61-2.47], *P* < .001).^15^ Additionally, a retrospective study in gastrointestinal cancer patients during the perioperative period revealed fewer adverse events associated with MLC than PICC, such as phlebitis (0.72 vs. 2.40%), bloodstream infections (1.08 vs. 2.88%), and thrombosis (0.72 vs. 3.37%).^16^ Our study aligns with these findings, revealing not only a low incidence of phlebitis but also favorable safety outcomes, with only one case each (1.7%) of catheter infection and DVT among cancer patients, a high-risk population that is particularly vulnerable to these conditions.

Patient-reported outcomes further support MLC as a preferred device for continuous 5-FU infusion. Our study showed that the majority of patients reported minimal to no symptoms of phlebitis, and over 90% expressed satisfaction with their experience of MLC use. Similar findings in a retrospective study revealed that patients undergoing perioperative cancer treatment reported higher satisfaction rates with MLC compared with PICC (69.5 vs. 51.9%).^16^ These findings suggest that improved comfort and relief from the anxiety of repeated venipunctures contribute to strong patient preference, indicating potential advantages of using MLC over other devices.

The extended MLC indwelling time, reported to be a median of 7 days (up to 49 days without replacement), contrasts sharply with PVC, which usually requires reinsertion within 3.5 days.^15,17^ Moreover, studies report that MLC is associated with lower occlusion rates than PICC (2.1 vs. 7.0%).^15^ In our study, only one case of occlusion (1.7%) was observed over a median indwelling time of 5.5 days, underscoring the durability of MLC use and the potential to alleviate healthcare providers’ workloads by reducing reinsertion demands. Additionally, the relatively low complexity of the MLC procedure and management reduces the need for specialized training, which facilitates nurse-led catheter insertion and management. These advantages were reflected in a survey of 56 participating physicians and nurses, in which the majority reported a reduced workload due to lower reinsertion frequency. Satisfaction levels were notably high, with 80% indicating they were “very satisfied” and 20% “somewhat satisfied.” These findings demonstrate the potential to promote broader MLC usage in clinical practice, contributing to more efficient healthcare resource allocation.

Several limitations of this study warrant consideration. First, this was a nonrandomized, prospective trial with a small sample size conducted at a single institution, focusing mainly on esophageal and head-and-neck cancer patients receiving continuous 5-FU infusion. Second, the analysis did not include direct comparisons with PVC or PICC, precluding any definitive conclusions regarding the relative advantages of MLC. Third, given the indwelling duration of the midline catheter, its utility may be limited when prolonged 5-FU administration is required, such as in palliative settings, as concerns arise regarding the need for frequent placements and associated costs. In such situations, a CV port would be a more reasonable choice. Therefore, MLC may be particularly useful in perioperative settings where 5-FU is administered over a defined period. Fourth, we used only one type of MLC, and so did not account for any variations across different devices, coatings, or manufacturers that may influence outcomes.

In conclusion, our findings suggest that MLC use offers a practical and an effective option for continuous 5-FU infusion, potentially improving patients’ QOL by reducing the incidence of phlebitis and lessening the burden on healthcare providers. Further comparative studies with other devices are encouraged to potentially expand the utility of this simple and accessible device within cancer care.

## Data Availability

Data will be shared on reasonable request to the corresponding author.
